# Explicit measurement of multi-tracer arterial input function for PET imaging using blood sampling spectroscopy

**DOI:** 10.1186/s40658-020-0277-4

**Published:** 2020-02-06

**Authors:** Carlos Velasco, Adriana Mota-Cobián, Jesús Mateo, Samuel España

**Affiliations:** 1 Centro Nacional de Investigaciones Cardiovasculares (CNIC), Madrid, Spain; 2 Departamento de Estructura de la Materia, Física Térmica y Electrónica, Facultad de Ciencias Físicas, Ciudad Universitaria, Universidad Complutense de Madrid, IdISSC, 28040 Madrid, Spain

**Keywords:** Arterial input function, Positron emission tomography, Multi-tracer PET, Gamma spectroscopy

## Abstract

**Background:**

Conventional PET imaging has usually been limited to a single tracer per scan. We propose a new technique for multi-tracer PET imaging that uses dynamic imaging and multi-tracer compartment modeling including an explicitly derived arterial input function (AIF) for each tracer using blood sampling spectroscopy. For that purpose, at least one of the co-injected tracers must be based on a non-pure positron emitter.

**Methods:**

The proposed technique was validated in vivo by performing cardiac PET/CT studies on three healthy pigs injected with ^18^FDG (viability) and ^68^Ga-DOTA (myocardial blood flow and extracellular volume fraction) during the same acquisition. Blood samples were collected during the PET scan, and separated AIF for each tracer was obtained by spectroscopic analysis. A multi-tracer compartment model was applied to the myocardium in order to obtain the distribution of each tracer at the end of the PET scan. Relative activities of both tracers and tracer uptake were obtained and compared with the values obtained by ex vivo analysis of excised myocardial tissue segments.

**Results:**

A high correlation was obtained between multi-tracer PET results, and those obtained from ex vivo analysis (^18^FDG relative activity: *r* = 0.95, *p* < 0.0001; SUV: *r* = 0.98, *p* < 0.0001).

**Conclusions:**

The proposed technique allows performing PET scans with two tracers during the same acquisition obtaining separate information for each tracer.

## Background

Positron emission tomography (PET) is a diagnostic molecular imaging technique that allows in vivo monitoring of metabolic processes within the body based on the biodistribution of a radiotracer that is administered to the patient. The wide variety of available radiotracers provides access to different biological aspects such as glucose metabolism, cell proliferation, hypoxia, or blood flow. Among the different radiotracers available, each one shows strengths and limitations for a particular clinical application [1]. Therefore, the nuclear medicine physician selects the tracer that will provide the most specific and reliable information for the patient under study. However, in many clinical cases, diagnostic accuracy can be increased considerably if complementary information is obtained from different tracers. An example of diagnosis using multiple tracers is found in ischemic heart disease, which includes evaluation of myocardial blood flow (MBF) using tracers like ^13^NH_3_, H_2_^15^O, or ^82^Rb and assessment of myocardial metabolism and viability using ^18^FDG. In this way, a better understanding of the pathophysiology of ischemic heart disease is obtained [2].

Conventional PET imaging has usually been limited to a single tracer per scan. Therefore, in order to perform PET examinations with multiple tracers on the same patient, different scans should be performed sequentially if the half-life of one tracer is short enough (i.e., tracers based on ^13^N, ^15^O, or ^82^Rb) to allow fast clearance of the tracer before the next tracer is administered. Otherwise, scans can be performed in different days. These procedures lead to extended scan time and to increased cost and complexity of patient management. Those limitations can be diminished by performing PET imaging on patients that have been administered with multiple radiotracers. However, multi-tracer PET imaging is still a challenging approach as annihilation photon pairs emitted from either tracer are indistinguishable. Therefore, some extra information is needed to disentangle the signal coming from each tracer.

Two main strategies have been proposed so far in order to enable the possibility of performing PET scans with multiple tracers simultaneously. The first approach uses dynamic imaging with staggered injections. In this case, a multi-tracer compartment model is used to separate the contribution from each tracer. However, different constraints must be applied on the kinetic behavior in order to separate each tracer contribution from the multi-tracer PET signal [3–5]. In the second approach, at least one of the injected tracers must be labeled with a radioisotope that emits a prompt gamma in addition to the positron, which can be detected in coincidence with the annihilation photons [6]. With this additional information, the signal coming from both tracers can be isolated by energy discrimination within the PET scanner. However, a relatively high (> 10%) branching ratio of the prompt gamma is required in this case, reducing the list of candidate radioisotopes to ^124^I or those with similar prompt gamma branching ratio.

In this study, we propose a new technique for multi-tracer PET imaging that uses dynamic imaging and multi-tracer compartment modeling including an explicitly derived arterial input function (AIF) for each tracer. For that purpose, PET studies should be performed with at least one of the co-injected tracers based on a non-pure positron emitter [7], i.e., which produces additional gamma emissions. In order to end up with a separate AIF for each radiotracer, blood samples are collected during the acquisition and further analyzed by gamma spectroscopy. Once separate AIFs are obtained, multi-tracer compartment modeling is applied to determine the kinetic parameters associated with each tracer. Using this methodology, no constraints to the kinetic behavior are required. In addition, clinically promising isotopes like ^68^Ga [8], which has a very low branching ratio for the extra gamma photons, can be combined with other regular isotopes like ^18^F. The proposed methodology was implemented and validated in pigs by a combination of two tracers for cardiac PET imaging, namely ^68^Ga-DOTA and ^18^FDG. While ^18^FDG is a very well-known tracer used in myocardial viability studies among other cardiac applications [9], ^68^Ga-DOTA has been recently proposed as a new PET tracer for MBF and extracellular volume fraction (ECV) determination [10–13] as well as for pulmonary blood flow [14].

## Methods

### Study design and experiment overview

**Fig. 1:**
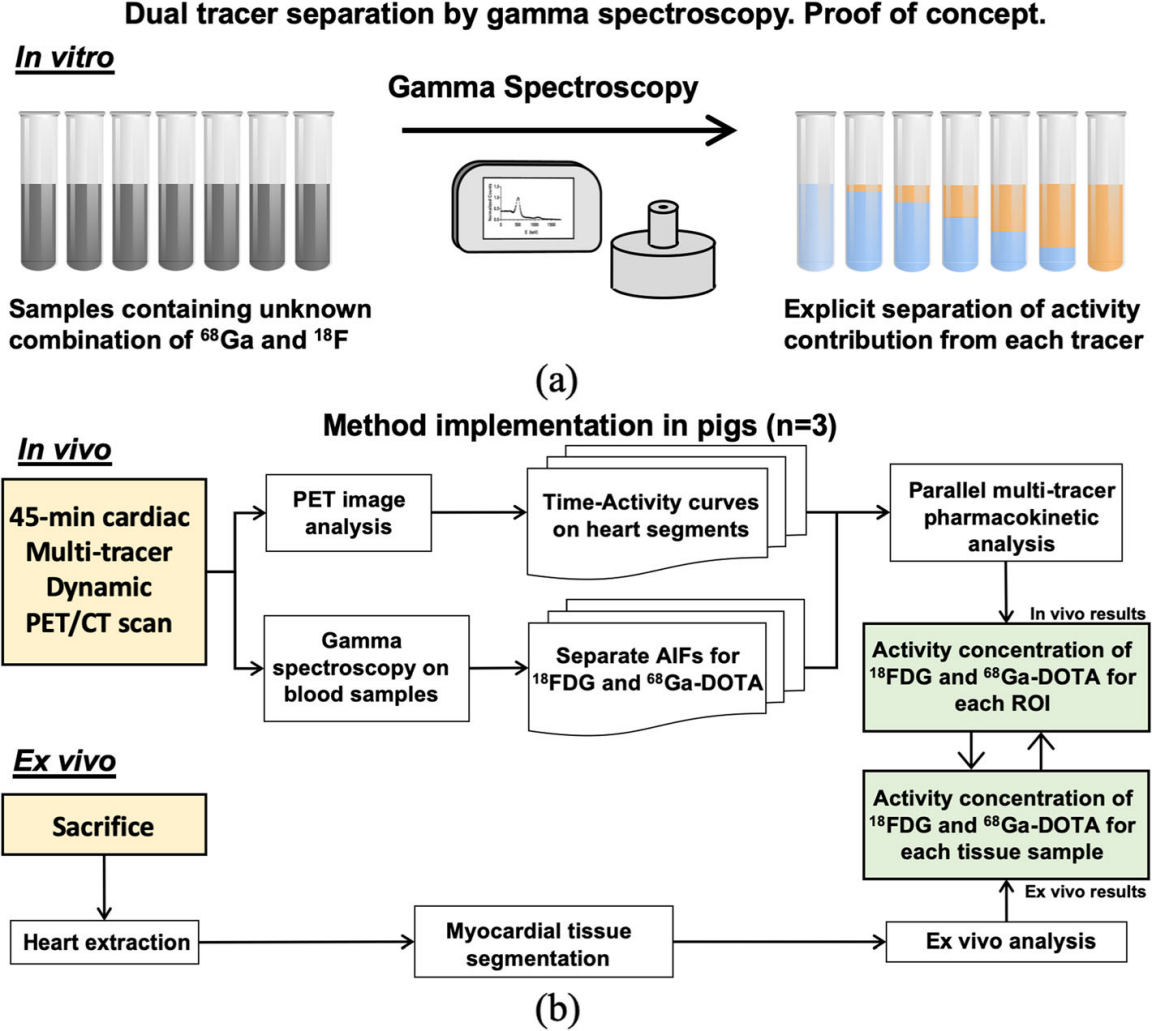
Schematic drawing of the study design. **a** Firstly, our proposed tracer separation methodology based on gamma spectroscopy was evaluated in vitro as a proof of concept. A calibration protocol was established to obtain the activity concentrations of each radioisotope for samples containing an unknown combination of ^18^F and ^68^Ga. **b** Afterwards, this methodology was implemented in vivo. To do so, three pigs underwent 45-min cardiac dynamic PET/CT scans in which ^68^Ga-DOTA and ^18^FDG were injected with a 5-min time gap. After PET/CT examinations, the animals were sacrificed, and their hearts excised, divided into segments, and analyzed to obtain the activity concentration of ^18^FDG and ^68^Ga-DOTA inside each segment. These results were compared against those obtained in vivo by parallel multi-tracer pharmacokinetic on the same regions of interest (ROIs) of their hearts. Explicitly separated AIFs needed for the pharmacokinetic analysis were obtained with our proposed method by gamma spectroscopy of a set of blood samples withdrawn during the scan

In the first place, in vitro studies were performed as a proof of concept of our proposed methodology. Samples containing unknown mixtures of two isotopes (^18^F and ^68^Ga) were analyzed by means of gamma spectroscopy. Several calibration procedures were carried out in order to obtain the individual contribution of each tracer. For in vivo studies, ^18^FDG and ^68^Ga-DOTA tracers were administered to healthy pigs, and dynamic PET scans were performed. Manual blood samples were collected throughout the PET scan and analyzed by gamma spectroscopy to obtain a separate AIF for each tracer. A multi-tracer compartment model was applied to the dynamic PET imaging using those explicitly separated AIFs. Finally, the model was used to determine the uptake of each tracer at the end of the PET scan on each segment of the myocardium, and the results were compared with those obtained ex vivo directly from myocardial tissue. To do so, animals were sacrificed, and the heart was excised in segments that were further analyzed to determine the individual uptake of each tracer ex vivo. A schematic drawing of the experimental protocol is shown in Fig. 1.

### In vitro tracer separation by gamma spectroscopy

We analyzed different combinations of two tracers, one based on a pure positron emitter (^18^F) and the other one based on a non-pure positron emitter (^68^Ga). While ^18^F only emits annihilation photons of 511 keV, ^68^Ga emits additional photons, but only those emitted at 1.077 MeV have a significant contribution (3.22%). Therefore, a sample containing an unknown combination of both isotopes could be analyzed by means of gamma spectroscopy. To determine the concentration of each tracer in a sample, a methodology was developed using a well counter (Wallac 1470 Perkin Elmer, Waltham, MA, USA) configured to record events during 1 min at different energy windows simultaneously, one of them covering the entire energy spectrum (200–2000 keV, hereafter named as *W*_200–2000_) and the other one covering only high-energy emissions (900–2000 keV, hereafter named as *W*_900–2000_). Dead time correction and background subtraction were implemented but not decay correction due to the unknown isotope combination. The amount of ^68^Ga and ^18^F contained in the sample was derived using the ratio (*Q*_S_) between events recorded at *W*_200–2000_ and *W*_900–2000_ energy windows as explained below.

**Fig. 2:**
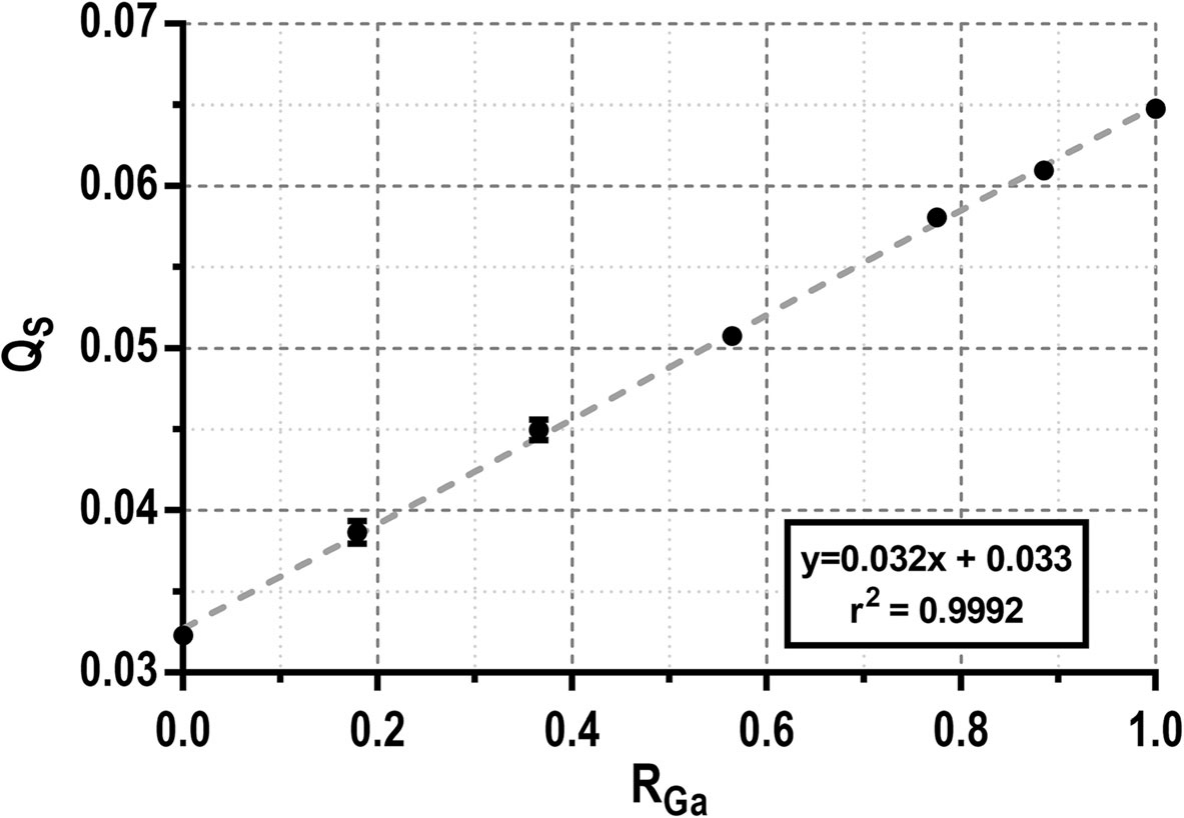
Calibration of *Q*_S_ values of a set of 1 ml samples with mixed ^68^Ga and ^18^F in different activity ratios. The linear fit (dashed line) shows an excellent linear correlation (*r*^2^ = 0.9992) between both datasets

**Fig. 3:**
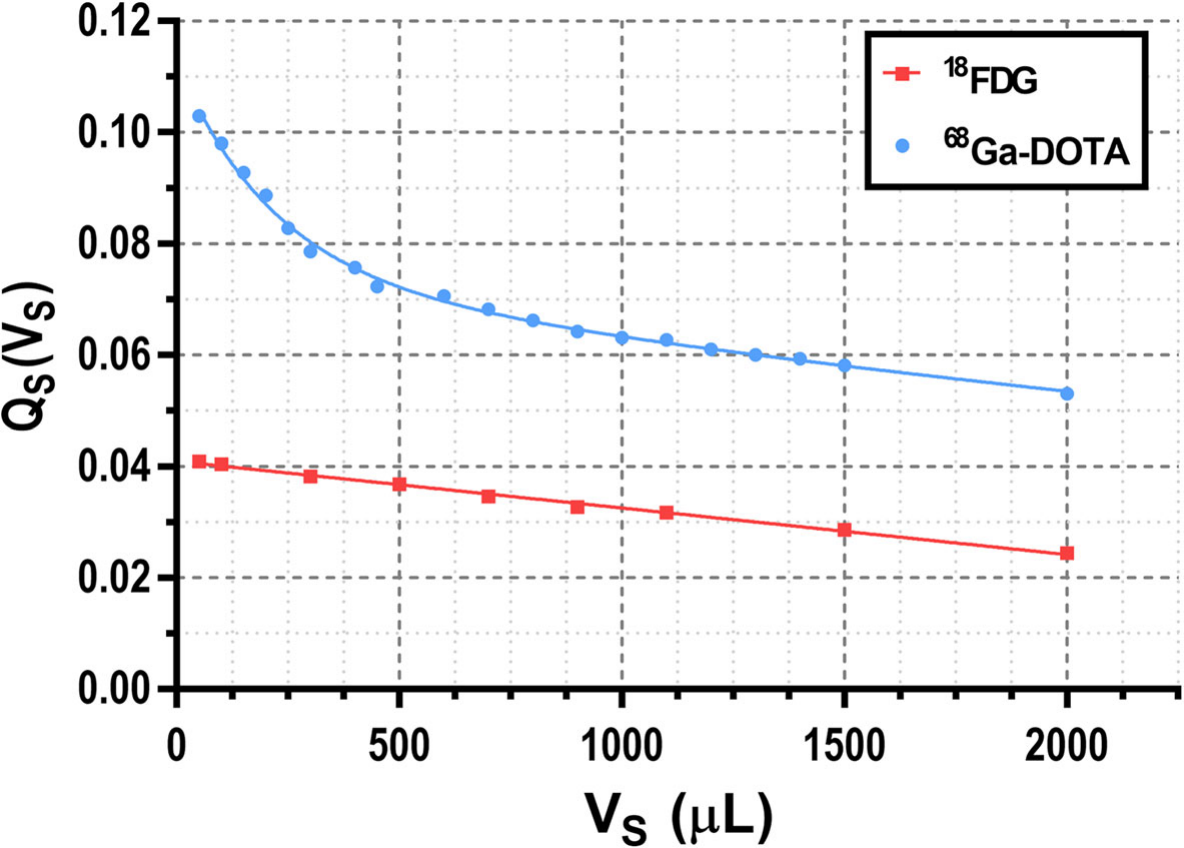
Variation of *Q*_S_ values measured with the well counter for pure ^18^F (red squares) and ^68^Ga (blue circles) with different sample volumes (*V*_S_). Results were fitted to a straight line and a sum of two exponentials respectively in order to obtain the *Q*_S_ values for different volumes

The relationship between *Q*_S_ and the relative activity of ^68^Ga (*R*_Ga_) and ^18^F (*R*_F_) in a sample (i.e., the individual fractional contribution of ^68^Ga and ^18^F to the total activity of the sample) was calibrated using a set of ^68^Ga/^18^F mixtures. Seven 1-ml samples were prepared containing ^68^Ga to ^18^F activity ratios 1:0, 9:1, 4:1, 3:2, 2:3, 1:4, and 0:1. These samples were analyzed in the well counter, and the *Q*_S_ values were represented against the known relative activities obtaining a linear relationship (see Fig. 2). Decay correction was applied to recorded values. Since in the subsequent animal studies, blood samples may be collected with different sample volumes (*V*_S_); a calibration had to be performed to account for variations in the detection efficiency of gamma photons with different energy and different geometrical distribution. For that purpose, *Q*_S_ values were recorded using pure ^18^F (*Q*_F_(*V*)) and ^68^Ga (*Q*_Ga_(*V*)) samples (~ 20 kBq each) with volumes ranging from 50 to 2000 μl (see Fig. 3). For known *Q*_F_(*V*) and *Q*_Ga_(*V*) within a sufficiently wide volume range, *R*_Ga_ and *R*_F_ can be obtained for a sample with known volume *V*s by solving the following equations:

1a $$ {Q}_{\mathrm{s}}\left({V}_{\mathrm{S}}\right)={R}_{\mathrm{Ga}}\cdotp {Q}_{\mathrm{Ga}}\left({V}_{\mathrm{S}}\right)+{R}_{\mathrm{F}}\cdotp {Q}_{\mathrm{F}}\left({V}_{\mathrm{S}}\right) $$

1b $$ {R}_{\mathrm{Ga}}+{R}_{\mathrm{F}}=1 $$

**Fig. 4:**
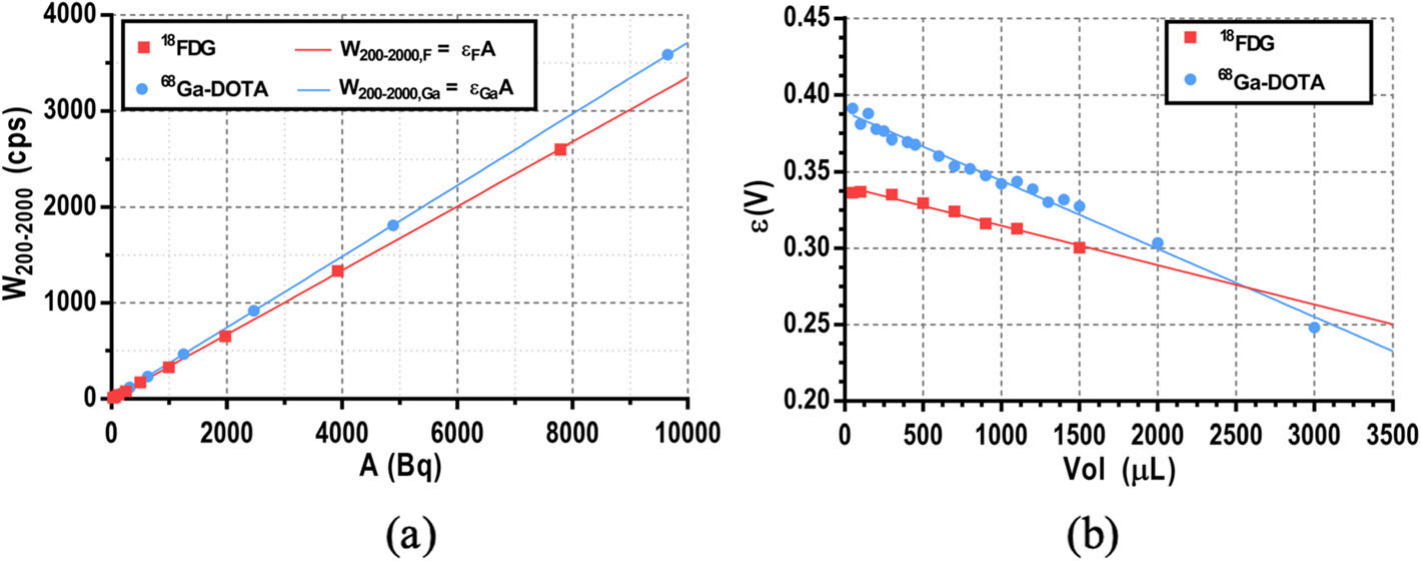
**a** Calibration profiles obtained in the well counter for 300-μl pure ^18^F (red squares) and ^68^Ga (blue circles) samples with different activity values using the full energy window (*W*_200–2000_). Each dataset was fitted to a straight line with *y*-intercept forced to be 0 obtaining the calibration factors *ε*_F_ = 0.335 cps Bq^−1^ and *ε*_Ga_ = 0.371 cps Bq^−1^ at this volume. **b** Variation of calibration factors with the sample volume for ^18^F (*ε*_F_) and ^68^Ga (*ε*_Ga_)

Afterwards, the absolute activity concentrations of each tracer (i.e., *A*_F_ and *A*_Ga_) can be obtained for a sample of known volume *V*_S_ as follows:

2a $$ {A}_{\mathrm{F}}\left[\mathrm{kBq}\cdotp {\mathrm{ml}}^{-1}\right]=\frac{A_{\mathrm{tot}}\cdotp {R}_{\mathrm{F}}}{V_{\mathrm{S}}}\kern0.75em $$

2b $$ {A}_{\mathrm{Ga}}\left[\mathrm{kBq}\cdotp {\mathrm{ml}}^{-1}\right]=\frac{A_{\mathrm{tot}}\cdotp {R}_{\mathrm{Ga}}}{V_{\mathrm{S}}}\kern0.75em $$

where *A*_tot_ is the total activity of the sample and can be derived from the following equation:

3 $$ {W}_{200-2000}={W}_{\mathrm{F}}+{W}_{\mathrm{Ga}}={A}_{\mathrm{tot}}\left({\varepsilon}_{\mathrm{F}}{R}_{\mathrm{F}}+{\varepsilon}_{\mathrm{Ga}}{R}_{\mathrm{Ga}}\right) $$

where *W*_F_ and *W*_Ga_ are the events recorded for each isotope, and *ε*_F_ and *ε*_Ga_ are volume-dependent calibration factors obtained from pure ^18^F and ^68^Ga samples respectively (Fig. 4).

Finally, before the kinetic model can be individually applied for each tracer, *A*_F_ and *A*_Ga_ were converted to β^+^decays·s^−1^ ml^−1^ multiplying by the branching ratio in order to match the units obtained from the PET images.

Following this procedure, explicitly separated AIFs can be obtained from a multi-tracer PET scan by analyzing a set of blood samples withdrawn from the subject throughout the study and obtaining *A*_F_ and *A*_Ga_ for each timepoint. The feasibility of this methodology was investigated in animal studies as described below.

### Animal protocol

The in vivo study was conducted according to the guidelines of the current European Directive and Spanish legislation and approved by the regional ethical committee for animal experimentation. Three healthy female white large pigs (mean weight = 45 ± 4 kg) were anesthetized by intramuscular injection of ketamine (20 mg/kg), xylazine (2 mg/kg), and midazolam (0.5 mg/kg) and maintained by continuous intravenous infusion of ketamine (2 mg/kg/h), xylazine (0.2 mg/kg/h), and midazolam (0.2 mg/kg/h). Oxygen saturation levels via pulse oximetry and electrocardiogram signal were monitored throughout the study. The coccygeal artery of the animal was cannulated and connected to a peristaltic pump placed as close as possible to minimize blood dispersion inside the tubing.

### PET/CT image acquisition

PET/CT images were acquired using a Gemini TF-64 scanner (Philips Healthcare, Best, The Netherlands). Each imaging study consisted of a low-dose CT scan (120 kV, 80 mA) followed by a dynamic 45-min list mode PET acquisition in a single bed position covering the entire heart. ^18^FDG (155 ± 12 MBq) and ^68^Ga-DOTA (142 ± 33 MBq) were injected 1 and 6 min after PET scan was started respectively. Both radiotracers were prepared in 6 ml and infused at a rate of 1.0 ml/s through a peripheral ear vein, followed by a 6-ml saline flush at the same rate. Arterial blood was withdrawn during the PET scan through a 1.6-mm internal diameter peristaltic pump tubing (TYGON-XL6, Saint-Gobain, Courbevoie, France) at 5 ml/min for the first 7 min and then at 2 ml/min for the rest of the scan. Blood collection from the coccygeal artery started immediately after the first radiotracer injection and continued during the whole study. During the first 12 min, blood was collected into sample tubes according to the following scheme: 20 × 5 s, 8 × 10 s, 6 × 20 s, 24 × 5 s, 6 × 10 s, 6 × 20 s, and 4 × 30 s. After that, 11 more samples were collected for 1 min with 2-min gaps between consecutive samples. PET images were reconstructed with a voxel size of 4 mm × 4 mm × 4 mm using a 3D RAMLA reconstruction algorithm in 84 consecutive frames (1 × 60 s, 25 × 5 s, 8 × 10 s, 4 × 20 s, 24 × 5 s, 3 × 10 s, 5 × 20 s, 5 × 60 s, 4 × 120 s, 4 × 180 s, and 1 × 300 s, total scan time 45 min). Corrections for dead time, scatter, and random coincidences were applied as implemented on the scanner. Decay and branching ratio corrections were not applied as the amount of ^68^Ga and ^18^F on each voxel is unknown, and their values differ (*t*_1/2_(^68^Ga) = 67.77 min and *t*_1/2_(^18^F) = 109.77 min, *B*_r,Ga_ = 0.891 and *B*_r,F_ = 0.967). Therefore, reconstructed images were expressed as β^+^decays·s^−1^ ml^−1^.

### Separate AIF derivation from blood sample gamma spectroscopy

After each PET/CT examination, the vials containing the collected blood samples were centrifuged briefly to provide a reproducible geometrical distribution of the blood before performing the measurements in the well counter. The volume for each blood sample was determined as the weight difference between empty and filled vial and applying a blood density of 1.03 g/ml [15]. Then, the individual activity concentration of ^18^FDG and ^68^Ga-DOTA (*A*_F_ and *A*_Ga_) for each blood sample was calculated using (1–3). Consequently, the AIFs obtained from blood samples for each tracer (AIF_BS,F_ and AIF_BS,Ga_) were derived as time series of these values.

Delay and dispersion corrections were applied to AIF_BS,F_ and AIF_BS,Ga_ using the image-derived AIF (AIF_ID_) as this one lacks delay and dispersion. AIF_ID_ was obtained from an 8-mm diameter cylindrical volume of interest (VOI) drawn in the descending thoracic aorta over five consecutive slices of the dynamic PET images. Spill-out from the AIF was corrected normalizing to the activity measured inside a 10-mm-diameter spherical VOI placed inside the left ventricle averaged over the latest frames. Delay was corrected by maximizing the cross-correlation between AIF_BS_ (sum of AIF_BS,F_ and AIF_BS,Ga_) and AIF_ID_. In order to obtain dispersion-free AIFs, we assumed that at the moment of the second tracer injection (at time *t*_2_), the blood concentration of the first tracer was changing slowly and therefore did not suffer from dispersion. Thus, dispersion before *t*_2_ is corrected by using the AIF_ID_ as there is only contribution from the first tracer. After *t*_2_, we assume that AIF_ID,F_ and AIF_BS,F_ are equal, and dispersion-free AIF for the second tracer can be obtained by direct subtraction of AIF_ID_ and AIF_BS,F_. Therefore, dispersion-free AIF_F_ and AIF_Ga_ used for pharmacokinetic analysis can be derived as follows:

4 $$ AI{F}_{Ga}=\left\{{\displaystyle \begin{array}{l}\kern3.479999em 0,\mid t<{t}_2\\ {} AI{F}_{ID}- AI{F}_{BS,F},\mid t\ge {t}_2\end{array}},\kern1.44em AI{F}_F=\right\{{\displaystyle \begin{array}{l}\kern0.48em AI{F}_{ID},\mid t<{t}_2\\ {} AI{F}_{BS,F},\mid t\ge {t}_2\end{array}} $$

### Kinetic modeling and image analysis

**Fig. 5:**
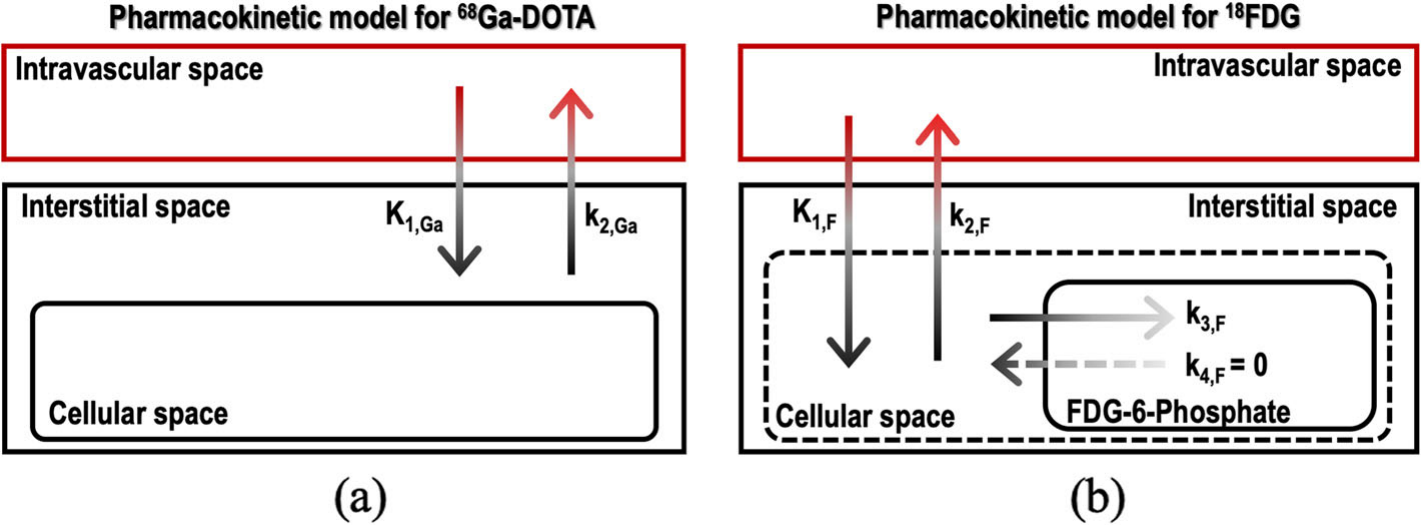
Kinetic compartment models for ^68^Ga-DOTA (**a**) and ^18^FDG (**b**). The model for ^68^Ga-DOTA is a single-tissue compartment model as the radiotracer diffuses bidirectionally between the intravascular space and extravascular extracellular space (interstitial space). The model for ^18^FDG is an irreversible two-tissue compartment model as the radiotracer diffuses bidirectionally between the intravascular and cellular space, and once it enters the myocyte, it can phosphorylate to ^18^FDG-6-phosphate and remains trapped as it cannot be further metabolized

Parallel multi-tracer compartment modeling [3, 5, 16, 17] was applied to the recorded PET data where each tracer’s kinetic behavior is introduced according to its pharmacokinetic model and to its individual AIF. ^68^Ga-DOTA diffuses bidirectionally between the intravascular and the interstitial space suggesting the use of a single-tissue compartment model (1TCM) [10, 12] (see Fig. 5a). On the other hand, ^18^FDG is explained with an irreversible two-tissue compartment kinetic model (2TCM) (Fig. 5b). Therefore, the total tracer concentration measured in the tissue (*C*_tis_) could be expressed as the sum contribution from both tracers:

5 $$ {C}_{\mathrm{tis}}(t)=\sum \limits_{\mathrm{i}=\mathrm{Ga},\mathrm{F}}{C}_{\mathrm{tis},i}(t)+\mathrm{PVE}(t)=\sum \limits_{i=\mathrm{Ga},\mathrm{F}}{\mathrm{IRF}}_i\left(\left\{{k}_{j,i}\right\},t\right)\otimes {C}_{\mathrm{p},i}(t)+\mathrm{PVE}(t) $$

where IRF_*i*_({*k*_*j*,*i*_},*t*) is the impulse response function for tracer *i*, {*k*_*j*,*i*_} are the kinetic parameters, *C*_p,*i*_(*t*) is the activity concentration in plasma for tracer *i*, and PVE(*t*) denotes the spill-over of radioactivity coming from LV and RV into myocardium. These IRFs can be described by the pharmacokinetic model that follows each tracer:

6a $$ {\mathrm{IRF}}_{\mathrm{Ga}}\left({K}_{1,\mathrm{Ga}},{k}_{2,\mathrm{Ga}},t\right)={K}_{1,\mathrm{Ga}}\cdotp {e}^{-{k}_{2,\mathrm{Ga}}\cdotp t} $$

6b $$ {\mathrm{IRF}}_{\mathrm{F}}\left({K}_{1,\mathrm{F}},{k}_{2,\mathrm{F}},{k}_{3,\mathrm{F}},t\right)={K}_{1,\mathrm{F}}\left(\frac{k_{2,\mathrm{F}}}{k_{2,\mathrm{F}}+{k}_{3,\mathrm{F}}}\cdotp {e}^{-\left({k}_{2,\mathrm{F}}+{k}_{3,\mathrm{F}}\right)t}+\frac{k_{3,\mathrm{F}}}{k_{2,\mathrm{F}}+{k}_{3,\mathrm{F}}}\right) $$

In order to obtain the free ^68^Ga-DOTA concentration in the plasma *C*_p,Ga_(*t*), hematocrit (*H*), and free metabolite fraction (*b*) must be used. These values have been previously determined [14]. On the other hand, ^18^FDG concentration in the plasma for myocardial tissue has already been described [18]. Therefore, the relation between AIF(*t*) and *C*_p_(*t*) for both tracers can be described as follows:

7a $$ {C}_{\mathrm{p},\mathrm{Ga}}(t)=\frac{b}{1-H}\cdotp {\mathrm{AIF}}_{\mathrm{Ga}}(t) $$

7b $$ {C}_{\mathrm{p},\mathrm{F}}(t)=\left(0.8+0.0012t\right)\cdotp {\mathrm{AIF}}_{\mathrm{F}}(t) $$

PVE contribution was not split for each tracer as it can be considered a function of the total blood activity concentration. It can be further decomposed in different components as follows:

8 $$ \mathrm{PVE}(t)={V}_{\mathrm{AP}}\cdotp {C}_{\mathrm{AP}}(t)+{V}_{\mathrm{LV}}\cdotp {C}_{\mathrm{LV}}(t)+{V}_{\mathrm{RV}}\cdotp {C}_{\mathrm{RV}}(t) $$

where *V*_LV_, *V*_RV_, and *V*_AP_ represent the spill-over fraction for the central LV, RV, and apical LV respectively [19], and *C*_LV_, *C*_RV_, and *C*_AP_ represent the corresponding time-activity curves in those regions. The apical term was added to account for temporal differences observed between the central LV and the apical LV in swine hearts. The obtained kinetic parameters were not affected by the fact that decay correction was not applied to AIFs and *C*_tis_ functions as both are affected in the same way.

The model was applied on time-activity curves (TACs) obtained from PET images. For that purpose, the myocardium was segmented using available software [20] following the standard American Heart Association (AHA) 17-segment model [21], obtaining one TAC (*C*_tis_ in (5)) per segment. *C*_LV_ and *C*_AP_ were obtained from spherical VOIs drawn at the center (15 mm diameter) and apical (12 mm diameter) regions of the LV respectively, while VOI for determination of *C*_RV_ was manually drawn inside RV over 3–5 slices leaving a margin (> 5 mm) from the myocardium. The 5-parameter model described on (5–8) was used to fit the data from each myocardial segment with a constrained Levenberg-Marquardt algorithm.

### In vivo versus ex vivo myocardial tissue analysis

The concentration of both tracers at the end of the PET scan (*C*_tis,F_(*t*_end_) and *C*_tis,Ga_(*t*_end_)) was computed for each myocardial segment using (5). In addition, the corresponding relative activities (*R*_F,PET_ and *R*_Ga,PET_) as well as standardized uptake values (SUV_F,PET_ and SUV_Ga,PET_) were also derived in the same regions at the imaging endpoints, i.e., the values derived from the tracer distribution at the end of the PET scan. In order to validate these results, analogous measurements were obtained from myocardial tissue samples at the same regions of the same animals that had undergone the PET examinations.

For that purpose, each animal was sacrificed at the end of the PET scans, and the heart was excised and divided into 17 segments also following the AHA guidelines [21]. Each segment was further divided into 3 smaller portions to obtain triplicate measurements. These 51 samples were weighted and measured in the well counter. In order to increase the accuracy of myocardial samples analysis, the measurements in the well counter were performed several times for each sample for 15 h using the full energy window (*W*_200–2000_). Measurements were corrected for dead time and background. The counts recorded as a function of time were fitted to a sum of two exponentials in order to recover the contribution from each tracer as follows:

9 $$ {W}_{200-2000}(t)={W}_{\mathrm{F}}(t)+{W}_{\mathrm{Ga}}(t)={W}_{\mathrm{F}}\left({t}_0\right){e}^{-{\lambda}_Ft}+{W}_{\mathrm{Ga}}\left({t}_0\right){e}^{-{\lambda}_{Ga}t} $$

where *λ*_F_ and *λ*_Ga_ are the radioactive decay constants for ^18^F and ^68^Ga respectively, and *W*_F_ and *W*_Ga_ are the counts measured in the well counter from each isotope. *W*_F_(*t*_0_) and *W*_Ga_(*t*_0_) were fitted using (9) and converted to activity using the corresponding calibration factors (see Fig. 4). Activity values were decay corrected at sacrifice time (end of PET scan), and the ex vivo relative activities for ^18^FDG (*R*_F,ex vivo_) and ^68^Ga-DOTA (*R*_Ga,ex vivo_) were obtained. The results obtained on each myocardial segment were averaged over triplicate samples. SUV values were also derived and extrapolated to the imaging endpoints for each tracer (SUV_F,exvivo_ and SUV_Ga,ex vivo_).

The ^18^FDG relative activities derived from tissue samples (*R*_F,ex vivo_) and from multi-tracer PET imaging (*R*_F,PET_) were compared using Pearson’s correlation and the root mean square error (RMSE), which is defined as follows:

10 $$ \mathrm{RMSE}\left(\%\right)=\sqrt{\frac{1}{N}\sum \limits_{s=1}^N{\left({R}_{\mathrm{F},\mathrm{ex}-\mathrm{vivo}}^{\mathrm{s}}-{R}_{\mathrm{F},\mathrm{PET}}^{\mathrm{s}}\right)}^2}\cdotp 100 $$

where *s* is the myocardial segment, and *N* is the number of myocardial segments analyzed (*N* = 17). In addition, SUV values derived from multi-tracer PET imaging were compared with values obtained from excised myocardial segments. For any statistical analysis, data are expressed as mean ± SD unless otherwise stated.

## Results

### Tracer separation by gamma spectroscopy

The results of the calibration procedure performed to separate the contribution of ^18^F- and ^68^Ga-based tracers from blood samples containing a mixture of both tracers are presented here. Figure 2 shows a linear behavior (*r*^2^ > 0.999) between the relative activity for ^68^Ga (*R*_Ga_) of different ^68^Ga-^18^F mixture samples and the *Q*_S_ value measured in the well counter. Figure 3 shows these *Q*_S_ values for pure ^18^F and ^68^Ga samples with volumes ranging from 50 to 2000 μl. Of note, the well counter detection efficiency for the high-energy gamma photon emitted by ^68^Ga is relatively higher at low sample volumes probably due to geometric factors. When the sample volume is small, high energy events represent about 4% of the total counts for ^18^F samples while it raises up to 10% for ^68^Ga samples. *Q*_S_(*V*_S_) profiles for ^18^F and ^68^Ga were fitted to a straight line and a sum of two exponentials respectively in order to interpolate to any given sample volume. Figure 4a shows the calibrations performed to translate the measurements obtained in the well counter using the full energy window to activity (data shown for ^18^F and ^68^Ga). Data presented in Fig. 4a were obtained from 300-μl samples. However, the calibration factors are also volume dependent. Therefore, the calibration was repeated for different sample volumes to account for this effect (see Fig. 4b).

### In vivo validation of multi-tracer PET against tissue analysis

**Fig. 6:**
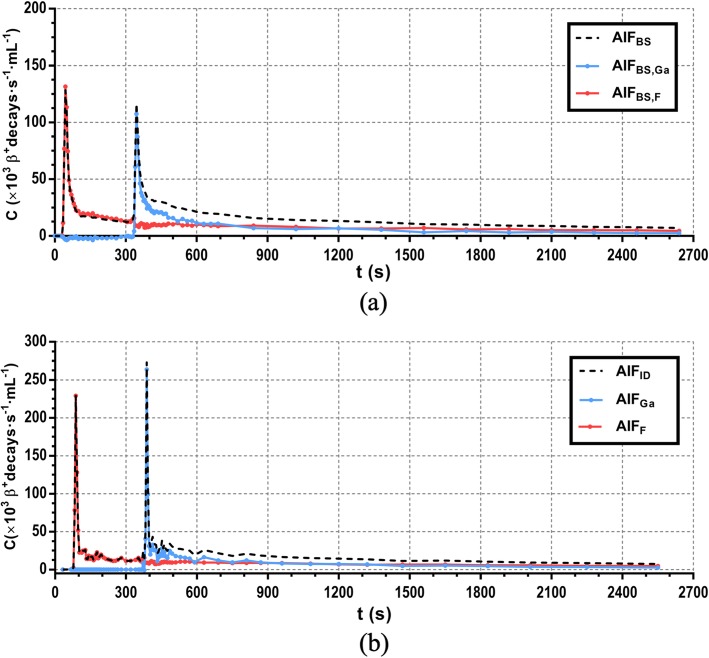
**a** AIF_BS,F_ (red) and AIF_BS,Ga_ (blue) obtained from manual blood sampling during PET scan applying the spectroscopic method. The black dashed line shows the sum of both tracers. **b** AIF_ID_ (black dashed line) obtained from the dynamic PET images using an ROI drawn in the descending thoracic aorta and delay corrected and dispersion-free contributions from ^18^FDG (red) and ^68^Ga-DOTA (blue) obtained as detailed in (4)

Figure 6a shows an illustrative AIF_BS,F_ and AIF_BS,Ga_ obtained from collected blood samples that were analyzed using the gamma spectroscopy methodology previously described. The corresponding AIF_ID_ is shown in Fig. 6b as well as the dispersion-free AIFs for each tracer (AIF_F_ and AIF_Ga_) which were obtained using the methodology explained in its corresponding methods section.

**Fig. 7:**
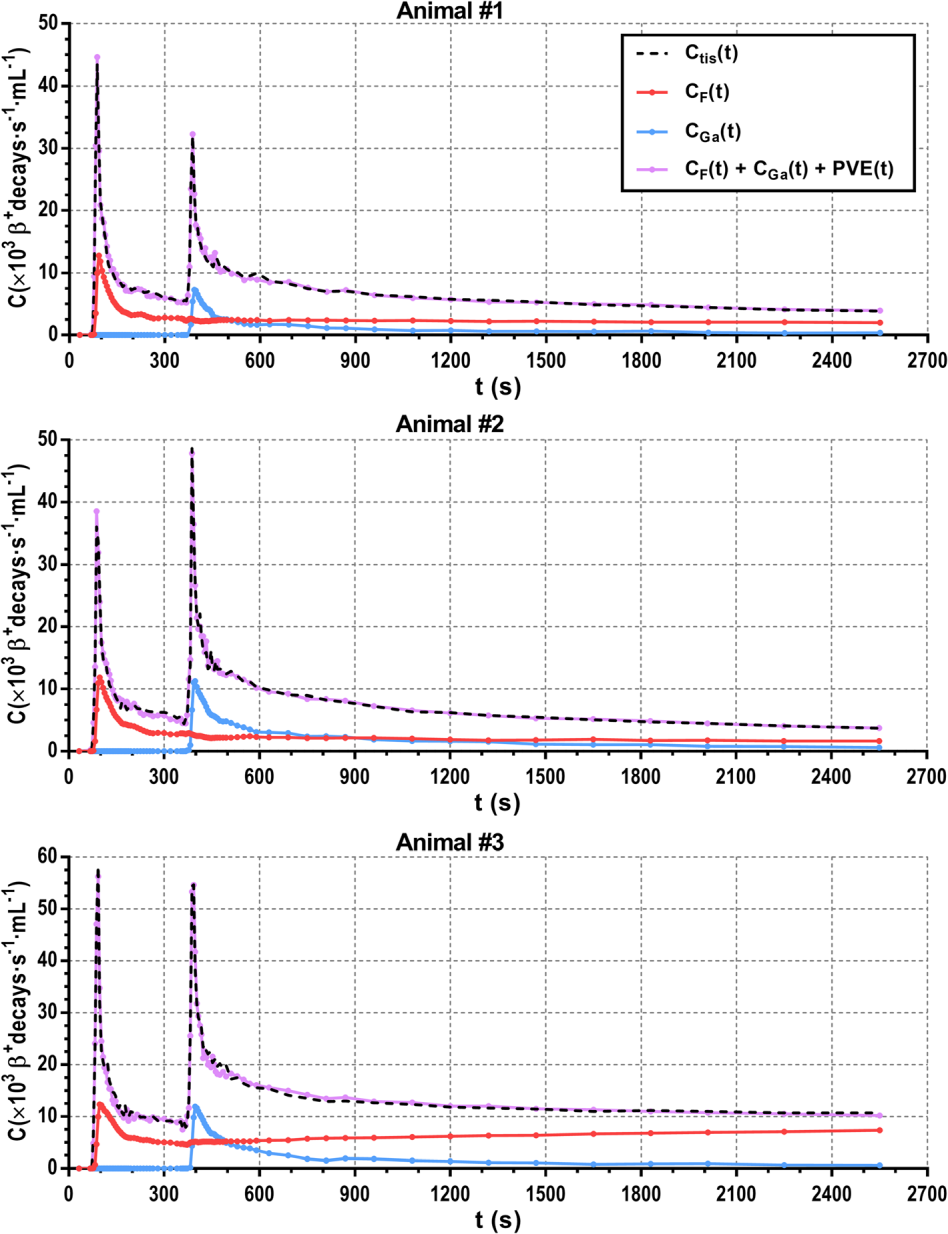
Myocardial tissue TACs obtained from dynamic PET images for each animal included in this study (black dashed lines). Data was fitted to the multi-tracer compartment model shown in (5) (purple line) and separated into tissue TACs for ^18^FDG (red) and ^68^Ga-DOTA (blue)

Figure 7 illustrates myocardial tissue TACs (*C*_tis_) obtained from dynamic PET data for each of the animals included in this study. These TACs were fitted using the multi-tracer compartment model shown in (5). The separate contribution obtained for ^18^FDG and ^68^Ga-DOTA is presented in Fig. 7 along with the total tissue signal including the spill-over.

**Fig. 8:**
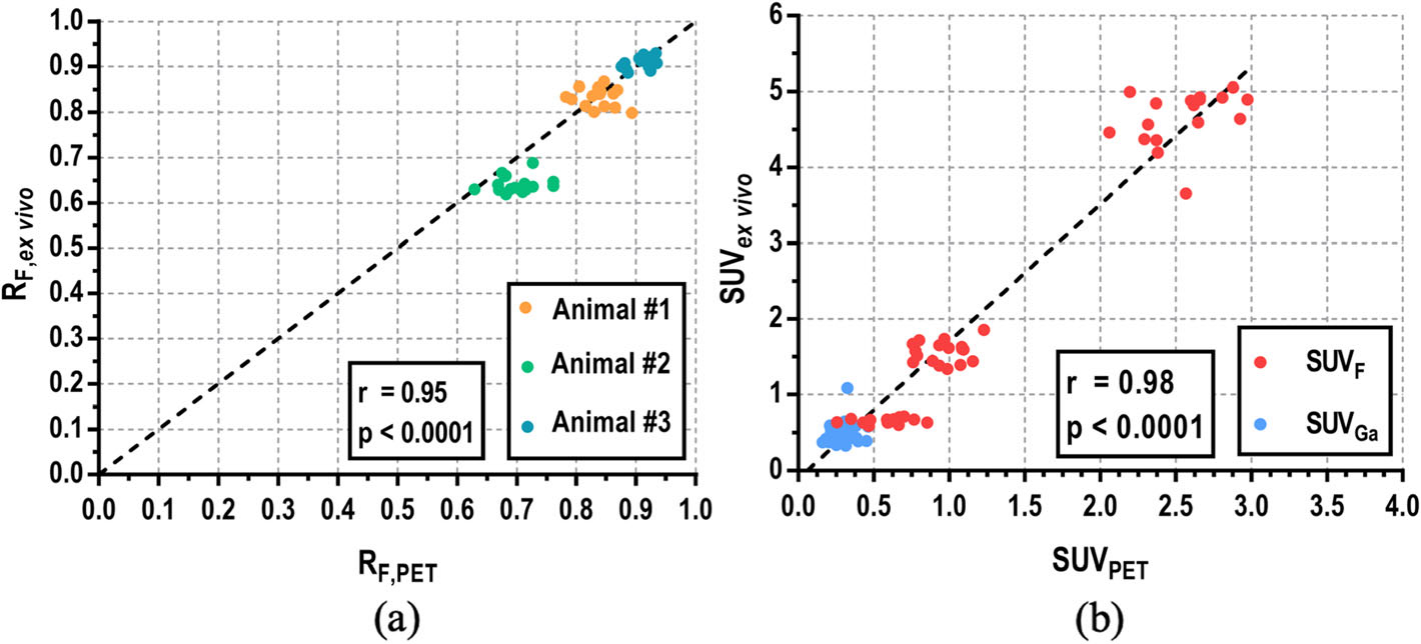
Linear correlation between relative activities for ^18^FDG (**a**) and between SUVs for both ^18^FDG and ^68^Ga-DOTA (**b**) obtained from multi-tracer compartment modeling at imaging endpoints (*R*_F,PET_, SUV_PET_) and from excised tissue (*R*_F,ex vivo,_ SUV_ex vivo_). Each dot represents one of the 17 myocardial segments for each animal. The results are highly correlated (Pearson’s *r* = 0.95, *p* < 0.0001 for relative activities and *r* = 0.98, *p* < 0.0001 for SUVs)

Figure 8a shows the comparison of the relative activity for ^18^FDG obtained from multi-tracer compartment modeling at imaging endpoints (*R*_F,PET_) and from excised tissue (*R*_F,ex vivo_) for each animal and myocardial segment. An excellent correlation was obtained (Pearson’s *r* = 0.95, *p* < 0.0001). Mean ± SD *R*_F,PET_ (*R*_F,ex vivo_) obtained were 0.84 ± 0.03 (0.83 ± 0.02), 0.70 ± 0.03 (0.64 ± 0.02), and 0.91 ± 0.02 (0.91 ± 0.01) for animals 1, 2, and 3 respectively. These averaged results, as well as RMSE and individualized SUVs for ^18^FDG and ^68^Ga-DOTA contributions, are presented in Table 1. SUV values obtained for ^68^Ga-DOTA were similar in all animals. SUV_Ga_ is low (~ 0.3) because this tracer reaches equilibrium between the plasma and the interstitial space, and therefore, the tracer does not accumulate in the tissue. On the other hand, low SUV_F,PET_ were obtained for animals 1 (0.97) and 2 (0.62) while higher values were obtained in the third animal (2.54). These SUV values are highly correlated (Pearson’s *r* = 0.98, *p* < 0.0001) with those obtained from excised tissue (SUV_F,ex vivo_ and SUV_Ga,ex vivo_). The lower RMSE value calculated for the third animal is consistent with the higher SUV_F_ obtained since higher uptake leads to lower statistical noise in the pharmacokinetic analysis, as well as in the measurements performed on excised tissue. In all cases, RMSE values were below 7%.

**Table 1:** *R*_F,PET_ and *R*_F,ex vivo_ values are represented as the mean ± SD of all the myocardial segments analyzed for each animal along with their comparison obtained using the RMSE value. Mean ± SD SUV for each tracer obtained from multi-tracer PET analysis and excised tissue are also shown

Animal	*R*_F,PET_	*R*_F,ex vivo_	RMSE (%)	SUV_Ga,PET_	SUV_Ga,ex vivo_	SUV_F,PET_	SUV_F,ex vivo_
1	0.84 ± 0.03	0.83 ± 0.02	3.6	0.30 ± 0.07	0.51 ± 0.09	0.96 ± 0.15	1.56 ± 0.14
2	0.70 ± 0.03	0.64 ± 0.02	6.8	0.27 ± 0.08	0.39 ± 0.03	0.60 ± 0.16	0.65 ± 0.03
3	0.91 ± 0.02	0.91 ± 0.01	1.7	0.28 ± 0.05	0.54 ± 0.06	2.55 ± 0.26	4.65 ± 0.36

## Discussion and conclusions

In this study, we proposed a novel technique to perform multi-tracer PET imaging using multi-tracer compartment modeling with explicit separation of individual AIF for each tracer. This technique relies on the use of two tracers with different isotopes with at least one of them being a non-pure positron emitter. If the energy of the additional gamma photons emitted by the non-pure positron emitter differs from the energy of annihilation photons, a spectroscopic analysis of blood samples containing both tracers can be performed in order to obtain the concentration of each individual tracer.

First, we developed a calibration procedure that allows the determination of individual tracer concentration of samples containing an unknown mixture of the isotopes used in this study (^18^F and ^68^Ga). For that purpose, samples were analyzed in a well counter recording event at two energy windows. The ratio between the counts recorded in both energy windows was later employed to determine the relative activity of each isotope. Corrections were made to account for different sample volumes (see Fig. 3).

The proposed technique was implemented in vivo by performing cardiac PET/CT studies on three healthy pigs, which were injected with ^18^FDG and ^68^Ga-DOTA during the same acquisition and validated against their analogous ex vivo measurements. A 45-min dynamic PET scan was performed on each animal, and blood samples were collected during the entire acquisition and further analyzed with the well counter to determine the AIF for each tracer. A multi-tracer compartment model was later applied to recover the individual tissue TAC for each tracer on individual myocardial segments (see Fig. 7). Imaging endpoint concentrations were validated against both ^18^FDG and ^68^Ga-DOTA concentration measured with the well counter on excised myocardial tissue. Results show that the proposed multi-tracer PET imaging technique offers very similar results to those obtained as a reference from ex vivo analysis (see Fig. 8), with RMSE below 7% in all cases. Moreover, SUV for ^68^Ga-DOTA and ^18^FDG was obtained showing normal ^68^Ga-DOTA uptake for healthy pigs [12] and variable ^18^FDG uptake as expected, since no prior glucose load was used [22]. An overestimation of SUV_ex vivo_ values compared with SUV_PET_ values can be observed, which might be explained by partial volume effect in PET data.

The proposed technique allows performing PET scans with two tracers during the same acquisition obtaining separate information from each tracer. This new method allows explicit measurement of separate AIF for each tracer while other existing methods rely on AIFs based on representative patients [23] or using extrapolation techniques [24]. However, our technique requires using at least one tracer based on a non-pure positron emitter, preventing the combination of tracers based on the same isotope. On the other hand, ^68^Ga is a suitable isotope for this technique. ^68^Ga has emerged as a very promising isotope for PET imaging with many relevant applications in clinical diagnosis [8]. Therefore, further combinations of tracers based on ^18^F and ^68^Ga, different from that shown in this study, might benefit from this work. Another limitation of our method is that it is invasive as it requires the collection of blood samples while other methods are non-invasive. Separation of AIFs could be generally applied using chromatographic techniques [25] although this is a very time-consuming process, and only a few blood samples could be analyzed.

Other techniques for multi-tracer PET imaging which use non-pure positron emitters with prompt gamma emissions record coincidence events with the PET scanner containing annihilation and prompt gamma photons, which are later used in the reconstruction process to separate the contribution from each tracer [6]. However, a higher branching ratio of the prompt gamma photons is required to obtain enough sensitivity of this type of events, and therefore, those techniques are limited to less common isotopes like ^124^I. It should be noted that in the proposed method, the additional gamma photons emitted by the non-pure positron emitter can be delayed with respect to the positron decay as they do not have to be detected in coincidence for the spectroscopic analysis.

In this study, the spectroscopic analysis of the blood samples was performed using a well counter. However, an online blood sampling detector [26, 27] could be also used, which would further simplify the implementation of this technique. We recently developed a novel blood sampling detector and successfully tested it in vitro for multi-tracer measurements based on the spectroscopic analysis [28]. In that case, the blood would be extracted from the patient through a catheter that would pass through a gamma photon detector, and a similar methodology to the one applied with the gamma counter would be used. In that way, AIF separation could be obtained immediately while minimizing the radiation exposure of the personnel and avoiding technical issues such as volume dependence of blood samples.

## Data Availability

Data and materials are available on request to the authors.

## Abbreviations

AIF: Arterial input function
FDG: Fludeoxyglucose
IRF: Impulse response function
MBF: Myocardial blood flow
PET: Positron emission tomography
PVE: Partial volume effect
RMSE: Root mean square error
SUV: Standardized uptake value
TAC: Time-activity curve
DOTA: 1,4,7,10-Tetraazacyclododecane-1,4,7,10-tetraacetic acid
